# Supplementary material for: The ATM Ser49Cys Variant Effects ATM Function as a Regulator of Oncogene-Induced Senescence
Source: Int J Mol Sci. 2024 Jan 29;25(3):1664. doi: 10.3390/ijms25031664 (PMC10855307; doi:10.3390/ijms25031664)
Supplement: Supplementary file 1 [file ijms-25-01664-s001.zip › ijms-2769884-supplementary.pdf]

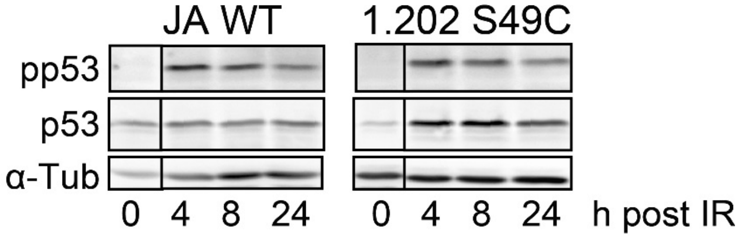


**Supplementary Figure S1:** The indicated LCLs were irradiated with 6 Gy IR then harvested at the indicated times. Whole cell lysates were immunoblotted for pp53, total p53 and α-tubulin (α-Tub) as a loading control.

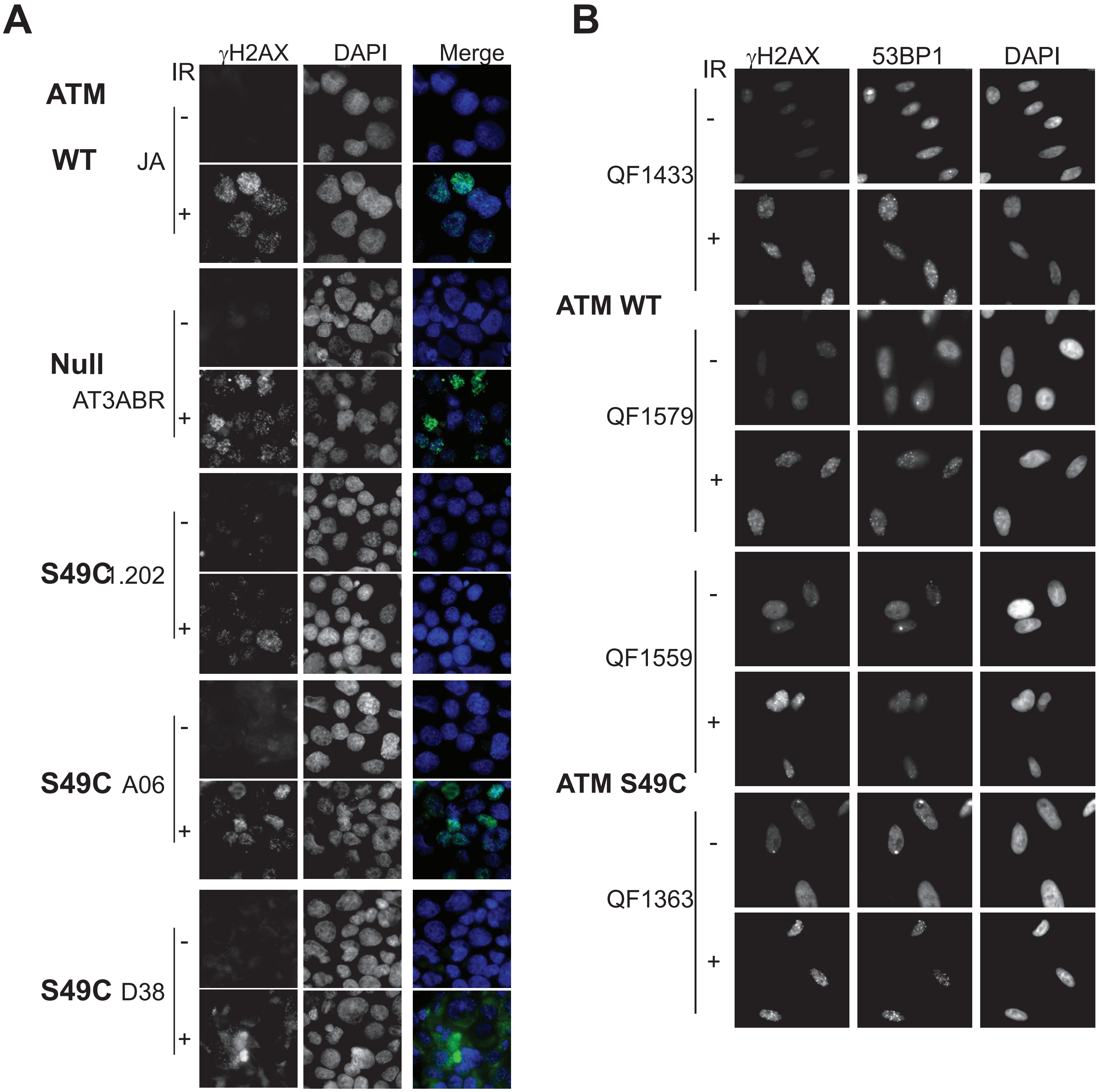


**Supplementary Figure S2**: Immunofluorescence staining of LCL (A) and melanoblast (B) cell lines. Cells were fixed 2 h with or without irradiation (6 Gy), and probed for γH2AX and 53BP1. DAPI stained the DNA.

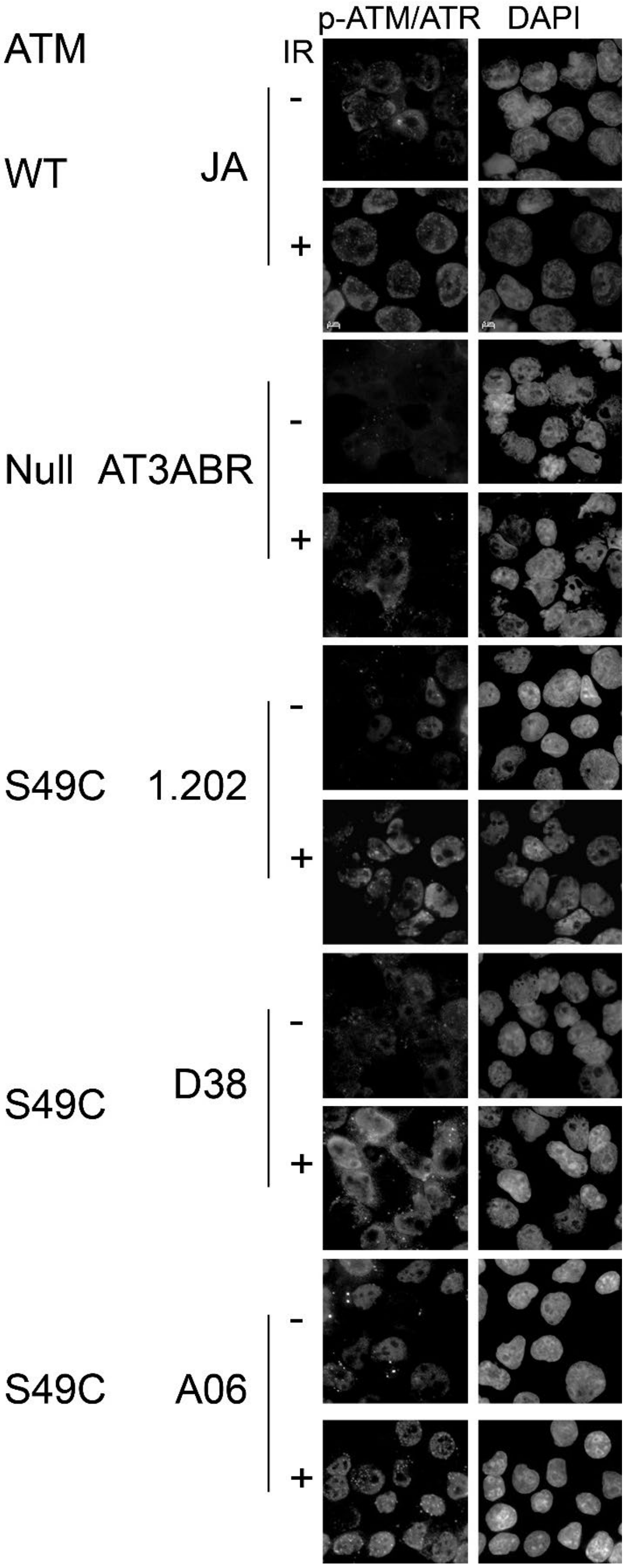


**Supplementary Figure S3:** Immunofluorescence staining of LCL lines. Cells were fixed 2 h with or without irradiation (6 Gy), and probed with the pATM/ATR substrate antibody, and DAPI for the DNA.

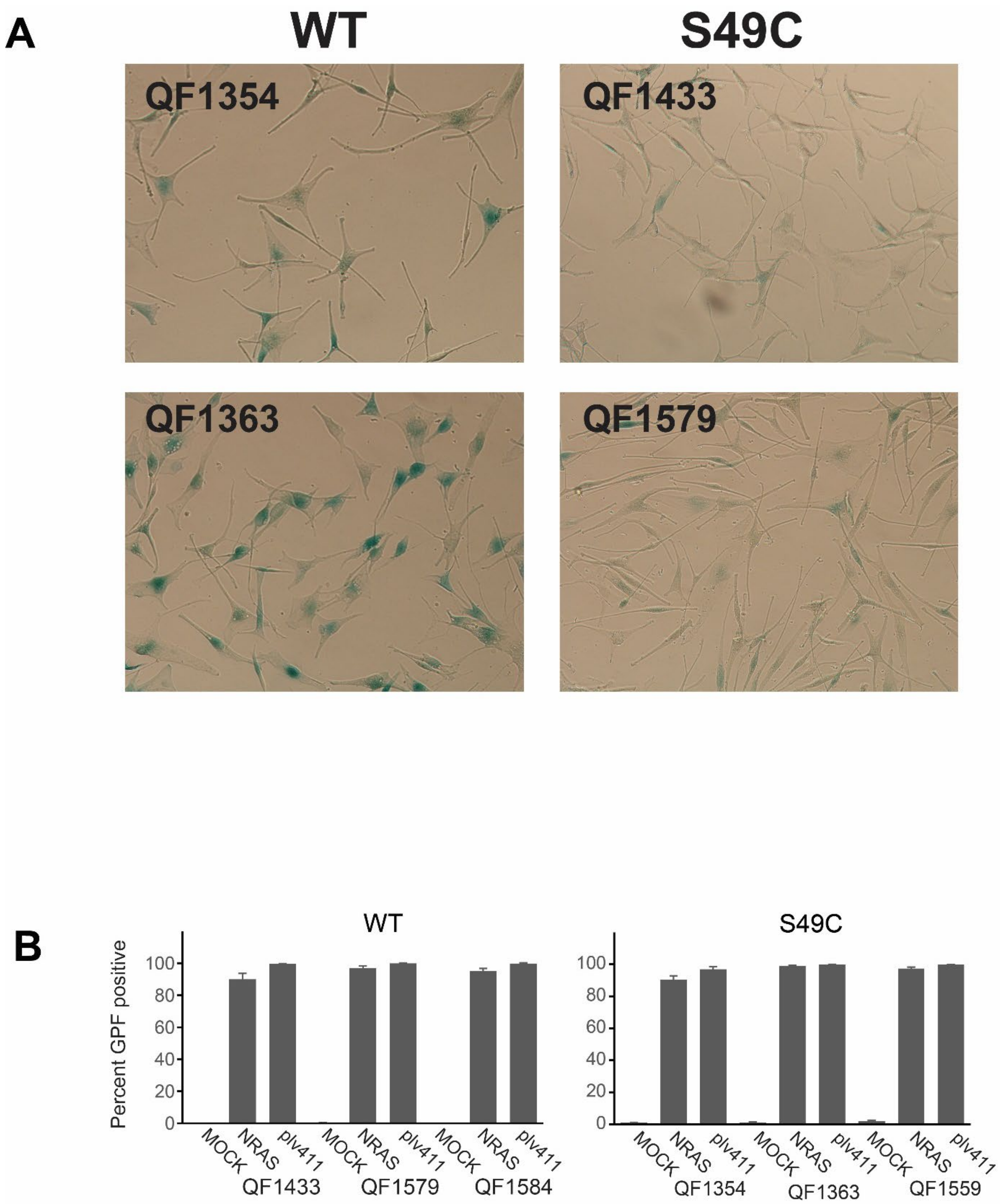


**Supplementary Figure 4:** A) Images of the indicated melanoblast lines 2 weeks after transduction with empty vector and stained for SA-β-Gal. B) High content image analysis of melanoblasts transduced with either empty vector (pLV411), NRAS Q61K or mock transduced for 2 weeks. The percentage of GFP expressing cells are shown. This is the average and SD counting 100 - 3400 cells per well of 6 wells.

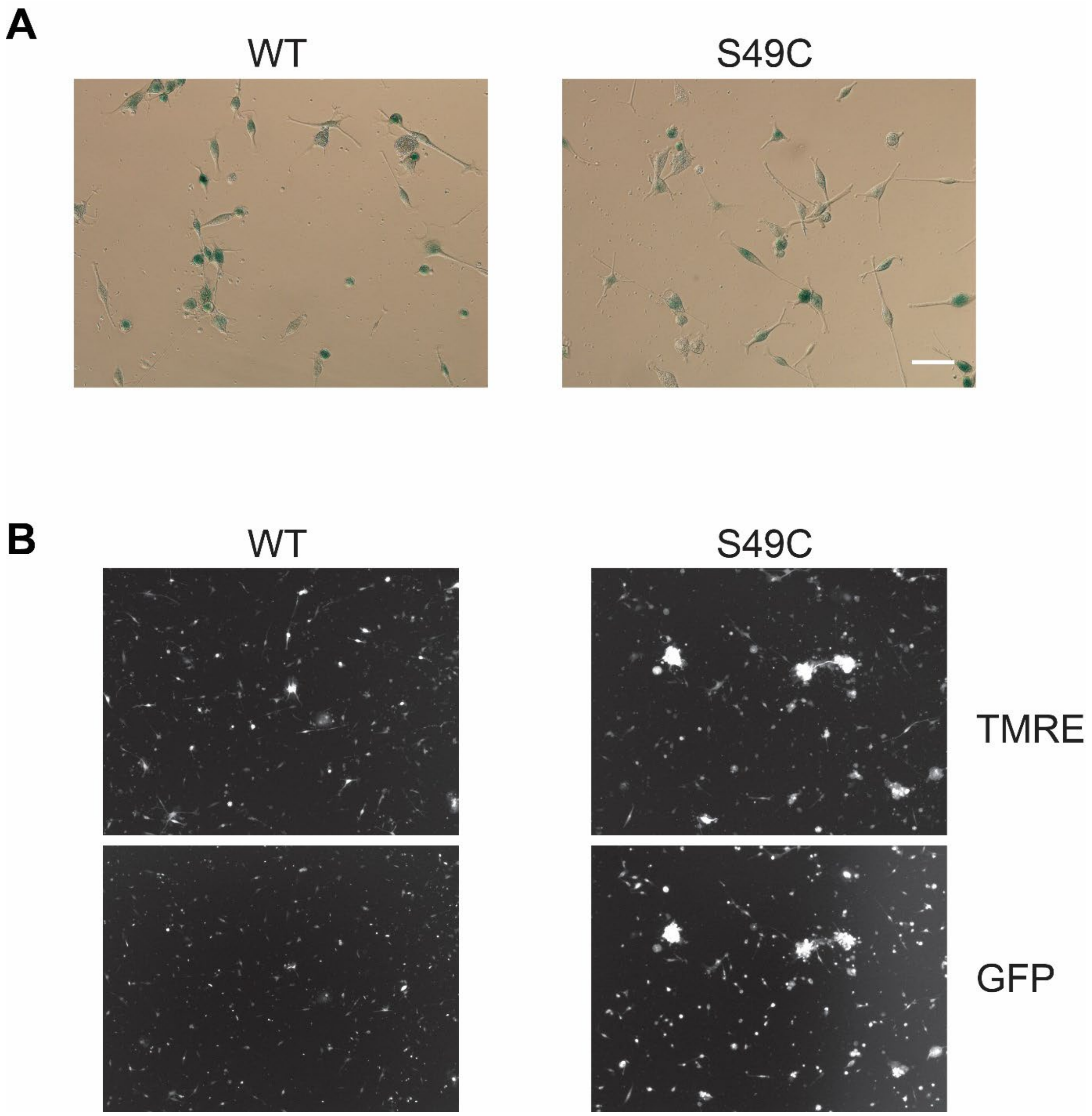


**Supplementary Figure S5:** A) Images of the indicated melanoblast lines 2 weeks after transduction with empty vector and stained for SA-β-Gal. B) Fluorescence imaging of the same cells as in Figure 5D but for GFP to identify the transduced cells and TMRE fluorescence for mitochondrial viability.

Supplementary Table S1: ATM Genotype of LCLs

| LCL | ATM | Mutation | State | Protein |
|---|---|---|---|---|
| JA | WT | | hom | WT |
| AT3ABR | ATM null | A8266T | het/- | K2756X |
| Col 1.201* | WT | | hom | WT |
| Col 1.202* | Variant | C146G | het | S49C |
| A06 | Variant | C146G | het | S49C |
| D38 | Variant | C146G | het | S49C |

* These LCL lines were derived from unaffected relatives of the proband of the melanoma and astrocytoma affected family [15] and genotyped as described in the Materials and Methods.

Supplementary Table S2: ATM Genotype of melanoblast lines used.

| QF # | MC1R | S49C |
|---|---|---|
| 1433 | WT | S/S |
| 1579 | WT | S/S |
| 1584 | WT | S/S |
| 1363 | D84E +/- | S/C |
| 1354 | R163Q +/- | S/C |
| 1559 | WT | S/C |
